# Thermodynamics and Intermolecular Interactions of Nicotinamide in Neat and Binary Solutions: Experimental Measurements and COSMO-RS Concentration Dependent Reactions Investigations

**DOI:** 10.3390/ijms22147365

**Published:** 2021-07-08

**Authors:** Piotr Cysewski, Maciej Przybyłek, Anna Kowalska, Natalia Tymorek

**Affiliations:** Department of Physical Chemistry, Pharmacy Faculty, Collegium Medicum of Bydgoszcz, Nicolaus Copernicus University in Toruń, Kurpińskiego 5, 85-950 Bydgoszcz, Poland; m.przybylek@cm.umk.pl (M.P.); 288310@stud.umk.pl (A.K.); n.tymorek@wp.pl (N.T.)

**Keywords:** nicotinamide, co-solvation, binary mixtures, heat capacity, fusion thermodynamics, COSMO-RS, DARE, intermolecular interactions, affinity

## Abstract

In this study, the temperature-dependent solubility of nicotinamide (niacin) was measured in six neat solvents and five aqueous-organic binary mixtures (methanol, 1,4-dioxane, acetonitrile, DMSO and DMF). It was discovered that the selected set of organic solvents offer all sorts of solvent effects, including co-solvent, synergistic, and anti-solvent features, enabling flexible tuning of niacin solubility. In addition, differential scanning calorimetry was used to characterize the fusion thermodynamics of nicotinamide. In particular, the heat capacity change upon melting was measured. The experimental data were interpreted by means of COSMO-RS-DARE (conductor-like screening model for realistic solvation–dimerization, aggregation, and reaction extension) for concentration dependent reactions. The solute–solute and solute–solvent intermolecular interactions were found to be significant in all of the studied systems, which was proven by the computed mutual affinity of the components at the saturated conditions. The values of the Gibbs free energies of pair formation were derived at an advanced level of theory (MP2), including corrections for electron correlation and zero point vibrational energy (ZPE). In all of the studied systems the self-association of nicotinamide was found to be a predominant intermolecular complex, irrespective of the temperature and composition of the binary system. The application of the COSMO-RS-DARE approach led to a perfect match between the computed and measured solubility data, by optimizing the parameter of intermolecular interactions.

## 1. Introduction

Nicotinamide (Niacin, NAM) is an important vitamin B_3_ constituent. It is used as a dietary supplement and medicine. The beneficial effects associated with the use of nicotinamide have been found, not only in diseases directly related to vitamin B3 deficiency (e.g., pellagra), but also in other diseases such as hyperlipidemia [1,2,3], hypercholesterolemia [4,5], and even depression and other mental illnesses [6,7,8]. Nicotinamide has also been found to exhibit antioxidant properties [9,10,11,12]. Due to its solubilizing properties and beneficial properties for health, nicotinamide is a popular pharmaceutical excipient used in cocrystals and co-amorphous compositions, exhibiting improved active ingredient dissolution behavior [13,14,15,16]. Nicotinamide’s dissolution behavior has already been extensively studied and some reports have been published quite recently. Some examples of neat solvents tested for their nicotinamide solubility at different temperatures are water, alcohols (methanol [17,18,19], ethanol [17,18,19], 1-propanol [19], 2-propanol [17,18,19], 1-butanol [17,19], isobutanol [19]), acetone [19], and esters [19] (methyl acetate, ethyl acetate, butyl acetate). When analyzing these data the nicotinamide solubility values expressed in molar fractions at 298.15 K can be ranked in the following order: water > methanol > ethanol > 1-propanol > 1-butanol > isobutanol > 2-propanol > acetone > methyl acetate > ethyl acetate > butyl acetate. This series clearly shows the advantage of protic polar solvents. Nevertheless, the available data in this comparison is not sufficiently diverse, and therefore it seems to be valuable to explore further the nicotinamide solubility in other solvents, including binary mixtures. Notably, the solubility of nicotinamide in methanol–ethanol and methanol-2-propanol was reported by Silveira et al. [18].

The solubility of organic compounds, and in particular drugs, in binary and ternary solvents has been a frequently explored issue, as evidenced by the numerous reports and reviews, including many works that have appeared in recent years [20,21,22,23,24,25,26,27,28,29,30,31,32]. Binary solvents have been successfully applied in various areas, including materials engineering, green chemistry technologies, and pharmacy. Some recently published reports provide interesting applications, for example a bioavailability enhancement of enzalutamide via spray drying technique using acetone-water binary solvents [33], lignin extraction from waste sawdust [34], the application of anti-solvent effects in perovskite solar cell manufacturing [35,36,37], and the supercritical fluid extraction of various phytochemicals such as phenolic compounds [38] and alkaloids [39]. The above mentioned applications demonstrate the usefulness of both solubility increase (co-solvation) and solubility decrease (anti-solvation). The main benefit of using mixed solvents is the ability to modify the properties of dissolution media by changing the proportion and composition of the solvent, which is very useful for designing media for important processes, such as the extraction and crystallization used in many branches of the chemical, food, and pharmaceutical industries. Binary aqueous mixtures deserve special attention due to the relatively good miscibility of water with many popular polar, moderately-polar, protic, and non-protic solvents; although some esters, ethers, higher alcohols, and hydrocarbons cannot be used due to problematic miscibility.

One of the many benefits of solubility studies is the development of theoretical models that can be used to optimize many processes such as extraction and crystallization. The aim of this study was threefold. First, the fusion thermodynamic properties of nicotinamide were analyzed by performing calorimetric and solubility measurements. The heat capacity change upon melting was measured and used for nicotinamide fusion quantification. The pool of available solubility data was extended by analysis of new aqueous binary mixtures containing both protic and aprotic solvents at different temperatures. Second, the affinity of nicotinamide for solvent molecules was quantified using advanced post-quantum chemical computations. Finally, the COSMO-RS-DARE (conductor-like screening model for realistic solvation—dimerization, aggregation, and reaction extension) methodology was applied for solubility prediction by direct inclusion of pairs representing the most stable structures at the saturated conditions. This method, although not used very often in the literature, is very promising, and it is worth confirming its effectiveness in the case of nicotinamide. To the authors best knowledge, this paper reports for the first time the adaptation of the DARE method for the study of solubility in mixed solvents.

## 2. Results and Discussion

Two important characteristics of nicotinamide fusion are discussed in terms of pure crystal properties and saturated solutions. These two interplaying aspects characterize the fusion thermodynamic properties of pure nicotinamide and those altered by non-ideal media. The crucial information, often omitted in studies focusing on solubility measurements, is the temperature trends of the heat capacities of the solid and melt states of solutes. This is justified by the fact that chemicals often sublimate or decompose below melting points, which prevents precise heat capacity measurements. Fortunately, this is not the case for nicotinamide, which enables a full and accurate characterization of fusion. It is worth mentioning that melting is reserved as a term for characterizing the phase change at a melting point, while fusion undergone at other temperatures is distinguished by using fusion as a more appropriate term.

### 2.1. Solid Characteristics

Nicotinamide crystalizes in stable solid in a monoclinic crystal form. Its crystal structure was solved as early as 1954 (CSD refcode NICOAM) [40], which was further confirmed by other single crystal X-ray measurements (CSD refcodes NICOAM01-09, excluding 04). Commercially available nicotinamide is supposed to be a native crystal form and stable under ambient conditions. However, under re-crystallization in some organic media the second form can appear (CSD refcode NICOAM04) [41]. This confirmed polymorphism of nicotinamide was anticipated prior to DSC measurements [42]. These polymorphic crystals differ greatly by the value of the melting temperature, since T_m_(I) = 397.8 K and T_m_(II) = 379.0 K [41]. Based on the values of the enthalpy of melting and melting points, it was found that polymorphs I and II are monotropically related [41]. Hence, polymorph I is supposed to be the thermodynamically stable form of nicotinamide between zero Kelvin and its melting point, while polymorph II is the thermodynamically metastable form. This remark is important, since the DSC measurements reported in this paper characterize the most stable nicotinamide crystal and no phase transition in the solid state was observed. Solid niacin has already been the subject of several thermochemical analysis [17,19,43,44,45,46,47,48,49,50], as summarized in Table 1.

It is worth commenting that the values of melting temperature are consistent with each other, with a mean value equal to 401.44 ± 1.36 K. The differences between measurements of melting enthalpy are slightly higher, with a mean value equal to 23.22 ± 2.89 kJ/mol. In this context, the results of measurements in this work are coherent with previously reported data.

The standard thermochemical characteristics were augmented with measurements of the values of the heat capacities of nicotinamide, both in the solid and melt states. These properties are indispensable for the adequate representation of the fusion properties in solubility models, including the activity coefficient determination in different solvents. Indeed, the mole fraction of the saturated solution can be directly related to the solid activity by the following fundamental relationship [51,52]:

(1) $$lnx_{eq}=lna_{eq}-ln\gamma_{eq}=lna_{s}-ln\gamma_{eq}$$

where the solid activity is related to the fusion Gibbs free energy $\Delta G_{}^{fus}\left(T\right)$

(2) $$lna_{s}=-\frac{\Delta G^{fus}}{RT}$$

The corresponding entropic and enthalpic contributions to fusion phenomena are related by the following fundamental relationship:

(3) $$\Delta G_{}^{fus}\left(T\right)=\Delta H_{}^{fus}\left(T\right)-T\cdot \Delta S_{}^{fus}\left(T\right)$$

Knowledge of the melting properties is required to actually take advantage of the above equations by the direct relationship of the fusion enthalpy to the relative value of heat capacity change upon melting,

(4) $$\Delta H_{}^{fus}=\Delta H_{}^{fus}\left(T_{m}\right)+\overset{T}{\underset{T_{m}}{\int }}\Delta C_{p}\left(T\right)dT$$

where $\Delta C_{p}^{fus}\left(T\right)=C_{p}\left(l\right)\left(T\right)-C_{p}\left(s\right)\left(T\right)$ stands for the temperature related difference between liquid and solid states. Analogically, the thermodynamic definition of the entropy of fusion $\Delta S_{}^{fus}\left(T\right)$ has the following functional form:

(5) $$\Delta S_{}^{fus}=\frac{\Delta H_{}^{fus}\left(T_{m}\right)}{T_{m}}+\overset{T}{\underset{T_{m}}{\int }}\frac{\Delta C_{p}\left(T\right)}{T}dT$$

**Figure 1:**
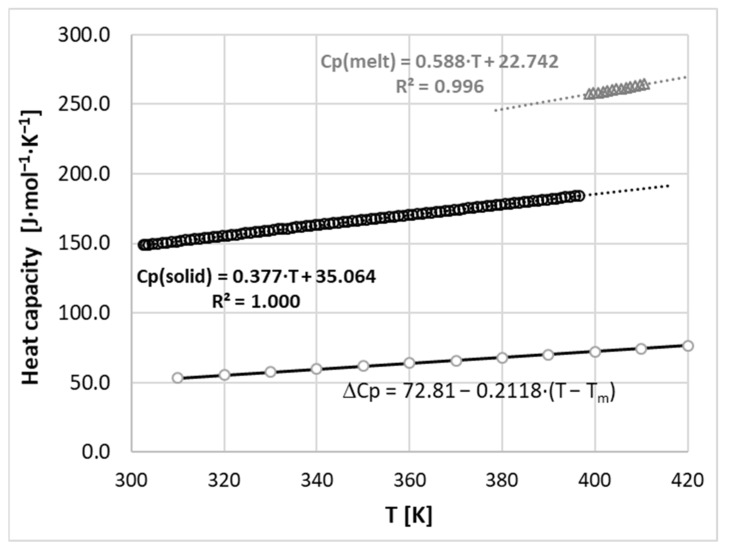
Measured temperature trends of solid and melt states of nicotinamide, where $\Delta C_{p}^{fus}\left(T\right)=C_{p}\left(l\right)\left(T\right)-C_{p}\left(s\right)\left(T\right)$ stands for the heat capacity difference between a supercooled liquid and solid.

**Figure 2:**
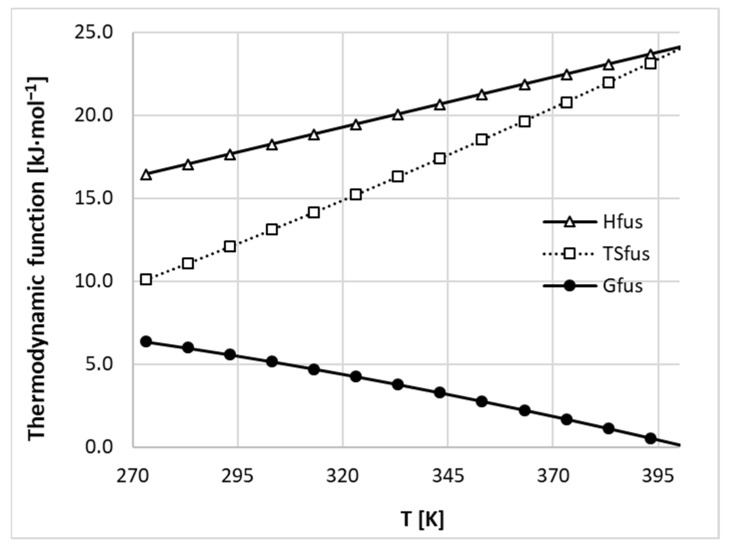
Temperature related changes of thermodynamic functions of nicotinamide fusion.

Hence, these fundamental relationships allow for a full temperature related thermodynamic characterization of the solid state. In Figure 1, the experimentally determined trends of heat capacities where plotted as a function of temperature. As one can see, the temperature change imposes fairly linear alterations to the heat capacity values. Taking advantage of this observation, a linear function was also attributed to $\Delta C_{p}^{fus}\left(T\right)$. The observed downward trend introduces a non-trivial correction for fusion enthalpy, which is more significant for the lower temperatures at which typical solubility measurements are performed. Taking advantage of Equations (3)–(5) and data provided in Figure 1, it is possible to fully describe nicotinamide’s thermodynamics in the solid state, as detailed in Figure 2. From the provided plots, it is clearly evident that the enthalpy contribution is more dominant at lower temperature ranges and dominates over the entropic contribution for all temperatures. At room temperate, the enthalpy/entropic compensation factor, $\left|\Delta H_{}^{fus}\right|$/$\left|TS_{}^{fus}\right|$, is equal to 1.42. Since temperature more seriously affects the fusion entropy term, the values of the Gibbs free energy of fusion become higher the further away the temperature is from the melting point.

### 2.2. Solubility Characteristics

**Figure 3:**
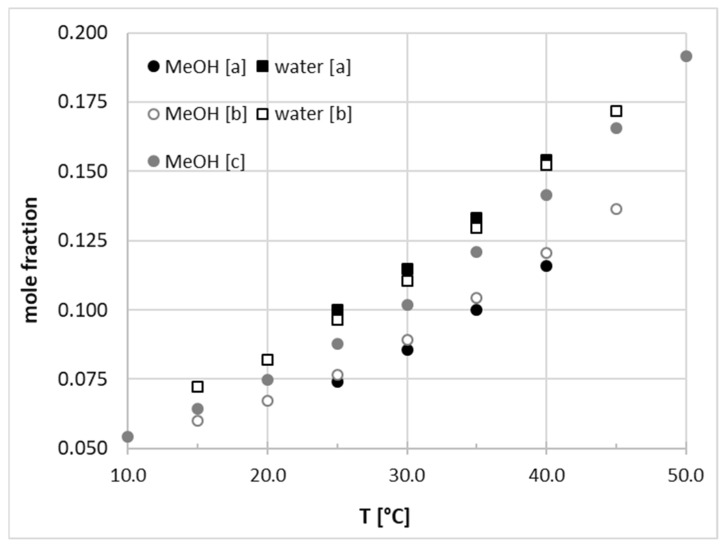
Solubility of nicotinamide in pure methanol and water estimated by this work (a) and according to ref. [17] (dataset (b)) and ref [19] (dataset (c)).

In this study the solubility of nicotinamide was determined for water, five neat organic solvents (methanol, 1,4-dioxane, acetonitrile, DMSO, DMF), and their aqueous binary mixtures. All measured data at four different temperatures (298.15, 303,15, 308.15, 313.15 K) are collected in Table 2 and Table 3. Additionally, graphical representations of the measured solubility data for all binary solvents are presented in Supplementary Materials in Figures S1–S5. It is worth mentioning that the dissolution data for neat methanol and water have already been published [17,19]. The comparison of these values with those reported herein is presented in Figure 3. From the provided plots it can be inferred that the data of this work match pretty well with those provided by Wu et al. [17]. However, there are some discrepancies between the measurements in this work and those published by Ouyang et al. [19].

As evidenced by the data provided in Table 2, two neat solvents, namely DMF and DMSO, are those in which nicotinamide dissolves in the highest quantities. When taking into account the literature solubility reported at 298.15 K [17,18,19] and the new values determined here, the following solubility series can be obtained: DMSO > DMF > water > methanol > ethanol > 1-propanol > 1-butanol > isobutanol > 2-propanol > acetone > 1,4-dioxane > methyl acetate > ethyl acetate > butyl acetate > acetonitrile. This shows that DMSO and DMF were found to be better solvents than water. Moreover, both these solvents in aqueous mixtures exhibit quite strong synergistic co-solvent effects. Interestingly, in the case of the DMSO–water binary mixture, the highest solubility advantage among all the considered solvents was observed with a 0.8 molar fraction of DMSO. In this case the solubility was about 2.6 times higher than the solubility in neat water and about 1.5 times greater compared to pure DMSO. On the other hand, the solubility of nicotinamide in acetonitrile was the lowest among all the considered solvents, and this solvent behaves like an efficient anti-solvent in the molar fraction range from 0.4–1.0. Finally, it was observed that the solubility of nicotinamide in methanol and 1,4-dioxane does not change much in comparison to water, and these solvent can be regarded as weak anti-solvents for nicotinamide. Notably, both 1,4-dioxane and acetonitrile were found to be characterized by a low nicotinamide solubility. Hence, it was found that the selected set of solvents for solubility measurements offered all sorts of solvent effects, enabling the flexible tuning of solubility that is so important for practical applications.

**Figure 4:**
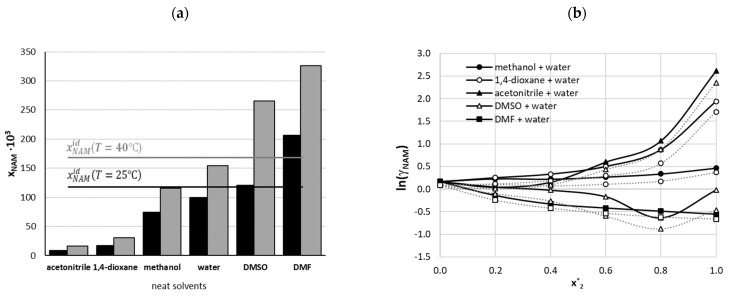
Characteristics of non-ideality of nicotinamide solubility in neat and binary solvents. (**a**) Comparison of ideal solubility measured at temperatures 25 °C and 40 °C; (**b**) solvent ratio dependent trends of activity coefficients of nicotinamide in studied binary solvents mixtures at 25 °C (solid black symbols) and 40 °C (grey open symbols).

It is interesting to see how ideal the neat and binary solutions are with respect to NAM solubility. This aspect is presented in Figure 4 by the comparison of ideal solubility with that measured in the neat solvents. Furthermore, the values of activity coefficients were determined for aqueous binary mixtures and plotted as a function of solvent ratio. From the data collected in Table 3 and Figure 4a it can be inferred that DMSO is closest to an ideal solvent of nicotinamide at room temperature. Interestingly, water can also be considered as a nearly ideal solvent of nicotinamide at ambient conditions, although the deviation is slightly higher compared to DMSO. The highest positive deviations from an ideal solvent are observed for DMF. On the opposite side, one can find acetonitrile, in which the solubility of nicotinamide at room temperature is about fourteen times lower compared to an ideal solvent. With a rise of temperature, deviations from ideal solubility are higher, but the above sequence of neat solvents is unchanged. The trends of activity coefficients varying with concentration change of organic solvent are provided in Figure 4b. Monotonous trends are observed for the majority of compositions of binary solvents. The only exception are DMSO–water systems at 0.8 mole fraction of this organic co-solvent. Hence, the nicotinamide activity in the whole range of concentrations of water–acetonitrile binary mixtures was the highest among all the systems studied here and significantly greater then unity. In the case of DMF, a systematic decrease of NAM activity is associated with the increase of the organic part in this aqueous mixtures.

### 2.3. Spectroscopic and DSC Characterization of Sediments

**Figure 5:**
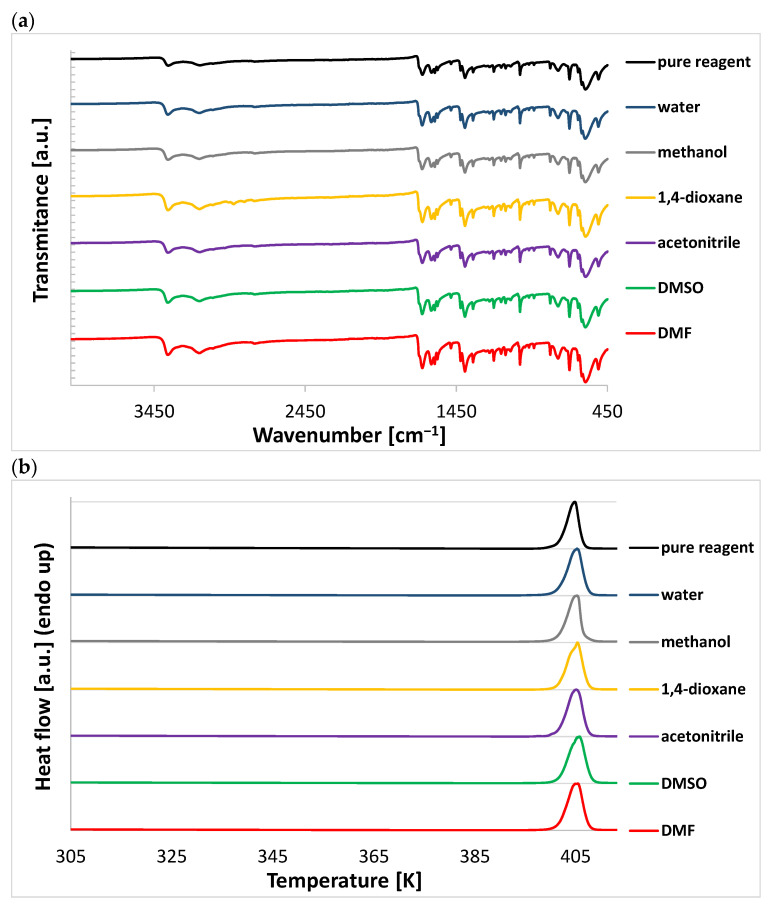
Results of the instrumental characteristics of pure nicotinamide and the sediments obtained after solubility measurements in pure solvents (**a**) FTIR-ATR spectra (**b**) DSC thermograms.

To complete the experimental part of the nicotinamide characterization and ensure that no solvates were formed and no polymorphic changes occurred during solubility measurements, additional instrumental analysis was undertaken. As one can see from both the IR spectra and thermograms collected in Figure 5, the plots characterizing different solid residues are almost indistinguishable from pure nicotinamide. In the case of the formation of a new molecular complex in the solid phase, such as solvate, shifts of the absorption bands corresponding to the polar groups are expected to occur. Pure nicotinamide spectra are characterized by the occurrence of symmetric and asymmetric NH stretching vibration bands located at 3149 and 3359 cm^−1^. Notably, a very slight deviations from these values (±2 cm^−1^) was found in the spectra recorded for the sediments. The DSC analysis indicated that no solvate decomposition or polymorphic transitions occurred, which is consistent with the conclusions drawn from the IR spectra.

### 2.4. Intermolecular Interactions of Nicotinamide in Aqueous Organic Solvent Mixtures

**Figure 6:**
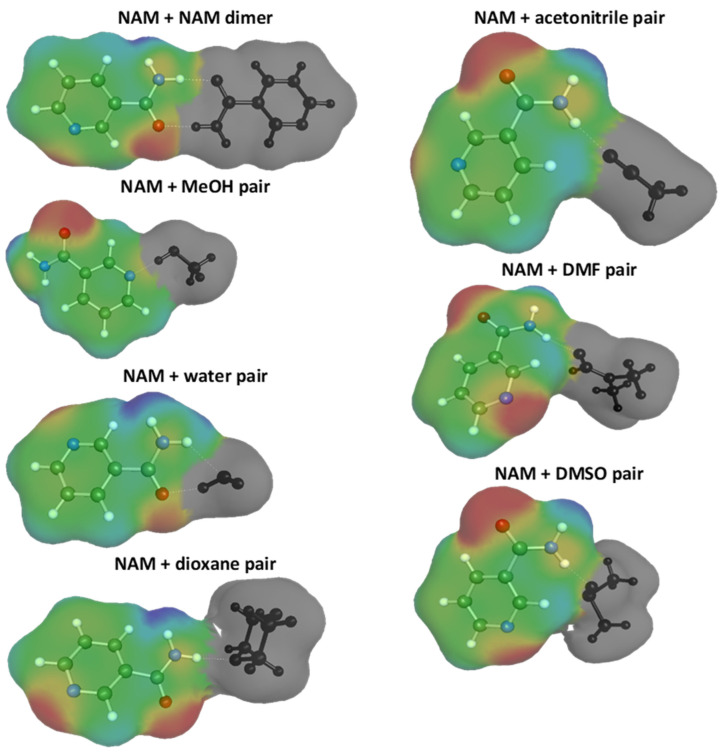
Graphical representation of pseudo-conformers included in the COSMO-RS-DARE computations of nicotinamide interactions with itself and each of the solvent molecules in the studied systems.

Application of COSMO-RS-DARE requires the identification of the most important intermolecular clusters, whose formation can occur due to the driving forces of intermolecular interactions. The analysis of the mutual affinities of solution components can be performed by computing the values of the Gibbs free energies of A + B = AB reaction. In this scheme, reactants represent nicotinamide and either of the binary solvent constituents, while the product is supposed to be in the form of a nicotinamide dimer or hetero-molecular complex of the solute with either of the solvent molecules. Identification of the corresponding structures allows taking into account the consequence of cluster formation on the possible intermolecular interactions in the saturated solutions. In the simplest case of no-pair formations, the only interactions possible are between monomeric forms of all constituents. On the other hand, the presence of any complexes affects the overall interaction pool, due to alterations of the properties of those species that are involved in intermolecular complex formation. This in turn has consequences for the macroscopic properties, including the chemical potential. In Figure 6 are presented structures of the most energetically favorable pairs identified through an extensive conformer search of possible contacts with various mutual orientations of interacting species. This graphical representation is provided in the format of the “mcos” files directly used in the COSMO-RS-DARE solubility computations. It is worth mentioning that there many more low energy clusters were found but only the ones identified as the most stable were included in Figure 6. The nicotinamide dimer is stabilized by an N-H∙∙∙O hydrogen bond motif formed by an $R_{2}^{2}\left(8\right)$ homosynthon and involving amide groups of interacting monomers. It is worth mentioning that both the experimental and theoretical calculations reported by Borba et al. [53] showed that the second type of hydrogen bond, with a heterocyclic nitrogen atom as acceptor, is much less preferred in the gas and amorphous glassy states. However, in the crystals, the stabilization contribution of the latter motif is significantly higher. The COSMO-RS calculations of the molecular complex structures correspond to the bulk liquid, and the obtained results are in good accord with former conclusions [53], suggesting that the most stable dimer shown in Figure 6 is more appropriate than the N-H∙∙∙N dimer representation. Interestingly, other hetero-molecular pairs included in Figure 6 were also inferred from NMR measurements [54]. In this context, it is worth commenting on the nicotinamide–water pair. In Figure 6, it is shown that the preferred complex identified by COSMO-RS was the one stabilized by a double hydrogen bond between the amide group and acceptor and the donor centers of water. The conclusion drawn from the chemical shift analysis [54] suggested the existence of an alternative pair stabilized with the hydrogen bond of water and with heterocyclic nitrogen acting as acceptor. Such a structure occurred in the pool of the most stable complexes computed using the RI-BP86 method, but it was found to be 3 kcal/mol less stable than the one shown in Figure 6. It should be emphasized that the inclusion of complexes exclusively in the form of pairs is a simplification, since more complex molecular assemblies are generally supposed to exist in bulk systems. However, even such a simple representation of complexes at the saturated conditions has been found sufficient from the perspective of solubility computations and the collection of all possible clusters potentially occurring in the analyzed systems is not necessary and is outside the scope of this paper.

**Figure 7:**
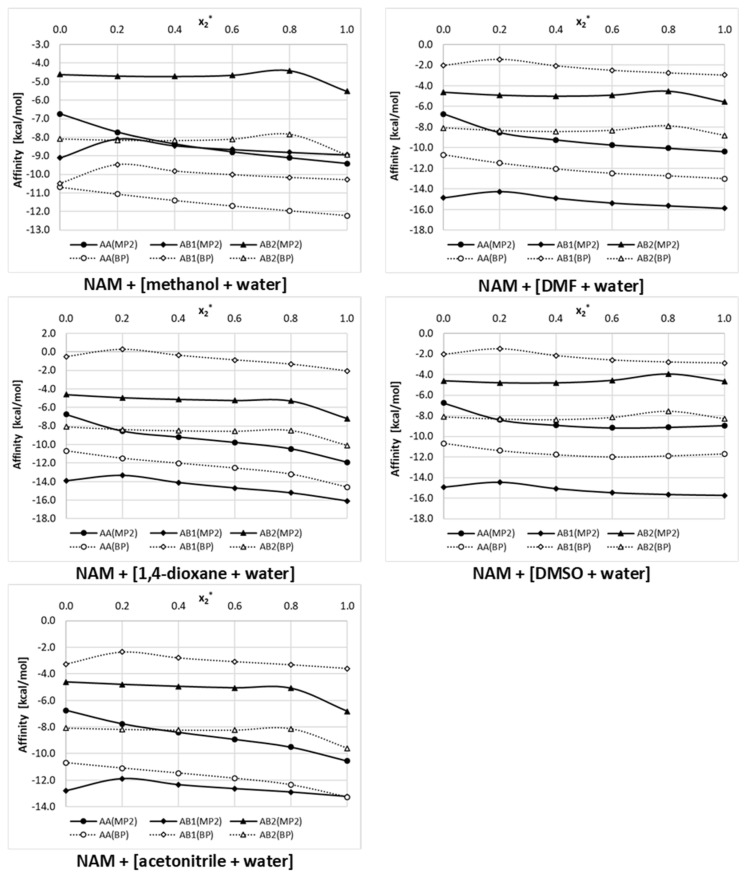
Concentration dependent nicotinamide affinity in aqueous binary solution of studied organic solvents (x_2_* denotes mole fraction of organic solvent in solute free solutions).

The thermodynamic properties of the whole population of pairs formed by nicotinamide are provided in Figure 7, where values of Gibbs free energies are plotted as a function of the varying composition of binary solvents at room temperature. For each system two series were determined, differing by the quantum chemistry method applied in the computations. All dotted lines represent results of the RI-DFT BP86 approach, and solid lines characterize data derived using the RI-MP2 method, which included corrections both for electron correlation and ZPE. The latter series are to be considered as more reliable according to Hellweg et al. [55]; recommendations suggesting the adequate methodology for a proper derivation of chemical equilibrium constants using quantum chemistry. It is interesting to note that there is serious discrepancy in trends derived using the two types of computations. In general, the DFT approach predicts lower affinities compared to the RI-MP2 computations. The only exception was found for the methanol + water system. Although both methods are consistent in their conclusion that all three types of binary complexes are stable, they disagree when the ordering of affinities of nicotinamide. Indeed, inclusion of the correction for ZPE and electron correlation reduces the probability of NAM–NAM dimers in favor of pairs comprising solute and organic solvent molecules. This observation is valid for all systems in the whole range of concentrations, except methanolic solutions in the diluted range of x_2_ < 0.5. The strong influence of the level of quantum chemistry computations on the predicted values of the Gibbs free energy of reactions involving solutes and solvent molecules has already been documented [56,57]. This suggests that final conclusions regarding the mutual affinities of studied systems should be drawn based on a more advanced level of computation. Hence, it seems that in all of the considered solutions all three types of complexes can occur, with the following order of decreasing affinity: NAM -organic solvent > NAM–NAM > NAM–water.

In Figure 7, the plotted trends of NAM affinities deserve some comments. Here, the affinity is synonymous to the values of the Gibbs free energy of the reaction of pair formation. This can be related either to the equilibrium constant expressed in the mole fraction (K_x_) or the activity (K_a_) consequently leading to ΔGr_x_ = −RTln(K_x_) and ΔGr_a_ = −RTln(K_a_). The former is concentration dependent, and the latter corresponds to a strict definition of the equilibrium constant and is concentration independent. The formal thermodynamic consistency is guarantee by utilizing values of activity coefficients apart from mole fractions. COSMOtherm offers computations of both of these data. In Figure 7, affinity is interpreted as Gr_x_ abbreviated by dropping the mole fraction subscript. This is why some deviations of affinity are noticed with the change of solvent mixture composition. It appeared that the influence of the solvent ratio was quite modest, although a non-linear concentration dependent trend was noticed. This might be related to the fact that any change in the composition of the binary solvent mixture is to be treated as new solvent with new physicochemical properties, including, among other things, the density, viscosity, polarity, and polarizability.

### 2.5. Results of COSMO-RS and DARE Computations

**Figure 8:**
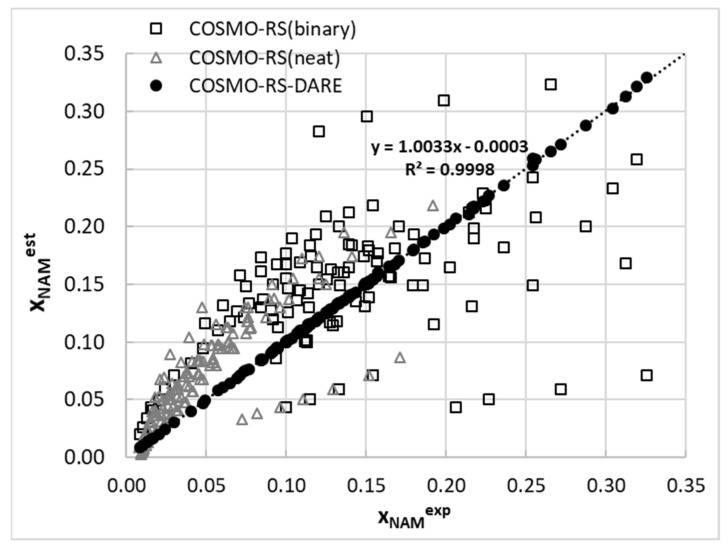
Predicted solubility of nicotinamide confronted with experimental data.

**Figure 9:**
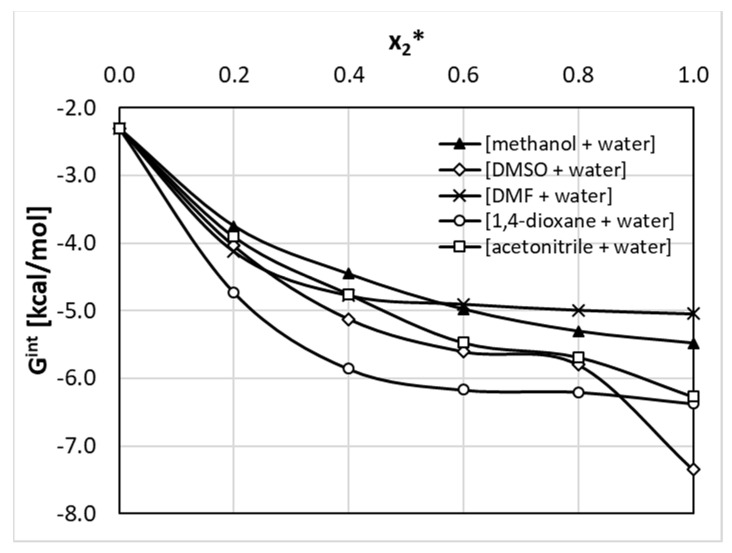
Concentration dependent distribution of optimized values of nicotinamide association enthalpy in the studied binary solutions (x_2_ * denotes mole fraction of organic solvent in solute free solutions).

The ability to compute solubility is highly desirable, not only from a theoretical point of view, but also from the perspective of practice in chemical and pharmaceutical technology. Unfortunately, to date no universal, accurate, and reliable theoretical approach has been developed, although there have been many partly successful proposals [58]. One of these is COSMO-RS, a promising in silico framework, enabling macroscopic property prediction from a molecular structure. Unfortunately, despite the clarity of formulation and some spectacular successes, in general a quite poor quality of prediction is achieved [59] in the case of solubility. This is also documented in Figure 8, where the distributions of computed nicotinamide mole fraction in a saturated condition and computed using the default COSMO-RS approach were confronted with the available experimental data. The results are only in qualitative agreement, and the inaccuracy is so high that the practical utilization of such predictions is prohibited. Such an unacceptable discrepancy between computed and measured solubility does not necessarily originate from drawbacks of COSMO-RS theory. It is partially expected that the model of studied solutions utilized in default solubility predictions will be inadequate. Indeed, if the intermolecular interactions responsible for the formation of complexes are taken into account, a new model emerges with species not considered in the default computations. This is why the DARE extension was considered here as a valuable tool for improving the accuracy of solubility computations. As documented in Figure 8, this is a highly successful approach, and an almost perfect match was obtained between the computed and experimental model fractions. Such spectacular success is achieved at the cost of introducing additional parameters defining intermolecular interactions. In Figure 9, these values are plotted as a function of the solvent composition at room temperature. Interestingly, the values of G^int^ monotonously decrease with the rise of organic solvent composition, with very small sensitivity to the temperature. This is why the plots corresponding to other temperature conditions are not provided. All trends were smooth and non-linear. The strongest interactions of nicotinamide seem to be in neat DMSO, followed by 1,4-dioxane and acetonitrile. It is worth noticing that interactions in DMSO are quite sensitive to water dilution, since a decrease of G^int^ is observed if the mole fraction of this organic solvent exceeds 0.8. The values presented in Figure 9 are to be treated as an additional set of parameters, which enables significant improvement of in silico computations of nicotinamide solubility in aqueous binary solvents. To the authors’ best knowledge this is the first trial of a COMSO-RS-DARE application for solubility computations in binary mixtures. The obtained results are very encouraging and worth further investigations.

## 3. Materials and Methods

### 3.1. Experimental Protocol

#### 3.1.1. Chemicals

All chemicals used in this study were of analytical grade and used without purification. Nicotinamide (CAS: 98-92-0) and 1,4-dioxane (CAS: 123-91-1) were obtained from Sigma-Aldrich (Poznań, Poland), while methanol (CAS: 67-56-1), acetonitrile (CAS: 75-05-08), dimethyl sulfoxide (DMSO, CAS: 67-68-5), dimethylformamide (DMF, CAS: 68-12-2), and sodium chloride were purchased from Avantor (Gliwice, Poland). For differential scanning calorimetry (DSC) measurements 99.999% nitrogen (Linde, Warsaw, Poland) was used.

#### 3.1.2. Solubility Measurements

The equilibrium solubility measurements were performed using the shake-flask method. A similar procedure has been utilized previously [21,25] and consists of the following steps: First, a mixture consisting of the solution with an excess of undissolved substance was prepared in 10 mL glass test tubes and shaken for 24 h at a controlled temperature using an Orbital Shaker ES-20/60, Biosan, Riga, Latvia(60 rpm). In all cases, the volume of solvent was 3 mL. In the next step, the suspended particles in the mixture were left to settle for an hour at the same temperature. Then, the liquid from above the sediment was collected by syringe and filtered using a 0.22 μm PTFE filter. Then, 500 µL of filtrate was withdrawn for density measurement using the pycnometric technique, while 100 µL was withdrawn for the solute concentration measurements. In order to avoid the risk of crystallization, the saturated solution used for nicotinamide concentration measurements was diluted immediately with 2000 µL of methanol. The syringe, filter, test tube into which the solution was filtered, and the pipette tip used in the diluting step were preheated in an incubator to maintain solubility measurement temperature conditions.

The concentration of nicotinamide in filtrates was determined by applying the spectrophotometric method. The calibration curve was performed using a absorption maxima at λ = 222 nm measured for nicotinamide methanolic solutions. The concentration range was from 0.239 to 0.077 mM. Due to the unmeasurably high absorbance, the samples were diluted with methanol during spectrophotometric measurements. The electronic spectra were recorded using an A360 UV-VIS spectrophotometer (AOE Instruments, Shanghai, China).

#### 3.1.3. FTIR-ATR Solid-State Characterization

Solid samples of nicotinamide were analyzed by FTIR-ATR measurements after collecting the sediments from the bottom of the tube after the flask-shake experiments. All samples were dried and measured using an FTIR spectrum two spectrophotometer (PerkinElmer, Waltham, MA, USA) equipped with a diamond attenuated total reflection (ATR) device. The spectrum of neat nicotinamide was also measured for comparison purposes.

#### 3.1.4. Differential Scanning Calorimetry (DSC)

In this study, DSC measurements were performed using a DSC 6000 Perkin Elmer (Waltham, MA, USA) device. The measurements were carried out in an inert atmosphere provided by a nitrogen flow (20 mL/min). The samples were sealed in aluminum pans. Prior to the measurements the calorimeter was calibrated using reference standards (zinc and indium) provided by the manufacturer. All measurements were performed three times to determine the average values of thermodynamic properties used for solubility modeling.

Thermograms were recorded applying both non-modulated and modulated techniques. In all cases, except the fast supercooling step, the heat flow rate was set to 5 K/min. The samples were weighed before and after each measurement, to confirm that there was no mass loss caused by the sublimation effect. Non-modulated measurements were used to determine the melting points (T_m_) and enthalpy of fusion (ΔH_m_) for pure nicotinamide and to characterize the sediments obtained after the flask-shake experiments. The modulated measurements were applied for determination of the heat capacity temperature profile of nicotinamide in solid and a supercooled liquid phases. The modulation period was 1 K, while the modulation amplitude was set to 120 s. In order to evaluate the reliability of the C_p_ measurements, a validation using sodium chloride was performed for the temperature range 300–500 K. The deviations from the reference values [60] did not exceed 2.4%. The relationship between the heat capacity and temperature for nicotinamide supercooled liquid was determined based on the approach described in works of Rasmuson et al. [61,62,63,64,65]. First, the sample was heated above the melting peak to 411 K, to ensure that all substance was melted. Then it was quickly cooled to 392.15 K, which is c.a. 10 K below the melting point. Since under these conditions, no exothermic peak corresponding to crystallization appeared during cooling step, a supercooled state was achieved. In order to determine the C_p_ values of the supercooled liquid, in the next step the sample was heated using a temperature modulation option.

### 3.2. Quantum-Chemical Computations

#### 3.2.1. COSMO-RS Computations

COSMO-RS (conductor-like screening model for real solvents), belonging to the class of quantum chemistry continuum solvation models augmented with statistical thermodynamics analysis of electrostatic surface interactions, offers direct calculation of the chemical potential of all components constituting the bulk phase. This combined approach is quite popular, so there is no need for repetition of the fundamentals, which can be found in the original publications [66,67,68]. Here, only a brief synopsis is provided. It is essential to state that the microscopic properties depicted though the quantum chemical part of this approach are related to macroscopic thermodynamic properties of a liquid by statistical thermodynamics of density probability distributions termed *σ*-profiles and *σ*-potentials. Integration of the latter over the surface leads to residual contribution to the chemical potential, allowing for predictions of almost all thermodynamic properties, including activity coefficients, excess properties, or partition coefficients and solubility. The quantum-chemistry part of COSMO-RS computes the interaction energy of a given molecule in a virtual conductor environment. In such an ideal conductor, the presence of the molecule leads to the induction of the polarization charge density, altering the solute–conductor interface. This in turn induces a response from the environment, back polarizes the molecular surface, and the quantum chemical computations are made to find the energetically optimal state of a conductor by altering the electron density until a self-consistency algorithm converges. This is also paralleled with the optimization of the molecular geometry in the vacuum. Once these steps are performed, which are the most time consuming, all the thermodynamic properties in any media can be computed using the second part of the COSMO-RS approach, by post-processing of the screening charge density. This represents the spatial distribution of the polarization density distribution in the form of a distribution–function termed *σ*-profile, p_i_(*σ*). This quantity signifies the histogram of the relative amount of surface with *σ*-density on the molecular surface. The *σ*-profile of multi-component systems is summed up with weighting by their mole fraction in the mixture. Based on this information, all intermolecular interactions are derived, including specific ones, such as electrostatic or hydrogen bonding, and non-specific, such as dispersion. The energy of the former is described as a function of the polarization charges of two interacting surface segments *σ* and *σ*′ in the following form:

(6) $$E_{MF}\left(\sigma ,\sigma^{\prime }\right)=a_{eff}\frac{a^{\prime }}{2}{\left(\sigma +\sigma^{\prime }\right)}^{2}$$

where 𝑎*_eff_* is the effective contact area between two surface segments and 𝛼′ is an adjustable parameter of the COSMO-RS approach. Furthermore, to account for the hydrogen bonding interaction, the energy function is defined based on the polarization charges of two interacting surface segments of acceptor, *σ_acc_*, and donor, *σ_don_*. Only such interactions are considered if two sufficiently polar pieces of a surface of opposite polarity are in contact. The following mathematical formula defines the quantity of these interactions:

(7) $$E_{HB}\left(\sigma ,\sigma^{\prime }\right)=a_{eff}c_{HB}\min\left(0;\min\left(0;\sigma_{don}+\sigma_{HB}\right);\min\left(0;\sigma_{acc}-\sigma_{HB}\right)\right)$$

where *σ_𝐻𝐵_* and *σ’_𝐻𝐵_* are adjustable parameters. The inclusion of Van der Waals (VdW) interactions in the COSMO-RS relies on the following definition:

(8) $$E_{vdW}\left(\sigma ,\sigma^{\prime }\right)=a_{rff}c_{vdW}\left(\sigma ,\sigma^{\prime }\right)=a_{eff}\left(\tau_{vdW}+\tau {\text{'}}_{vdW}\right)$$

where 𝜏_𝑣𝑑𝑊_, 𝜏′_𝑣𝑑𝑊_, and 𝑐_𝑣𝑑𝑊_ are element specific adjustable parameters. Hence, the vdW contribution to the total energy depends only on the type of atoms involved in the surface contact. These microscopic state properties characterizing the interaction between surface segments are related to macroscopic thermodynamic properties of a liquid by statistical thermodynamics. The integration of the *σ*-profiles over the surface leads to the chemical potential of a surface segment, $\mu_{s}\left(\sigma \right)$, by using the following formal definition:

(9) $$\mu_{s}\left(\sigma \right)=-\frac{RT}{a_{aff}}ln\left[\int P_{s}\left(\sigma^{\prime }\right)exp\left\{\frac{a_{aff}}{RT}\left[\mu_{s}\left(\sigma^{\prime }\right)-E_{misfit}\left(\sigma ,\sigma^{\prime }\right)-E_{HB}\left(\sigma ,\sigma^{\prime }\right)\right]\right\}d\sigma \text{'}\right]$$

The above value often called *σ*-potential is converted into the values of chemical potential of *i*-th compound in system S, $\mu_{S}^{i},$ by integration of the *σ*-potential over the whole molecular surface:

(10) $$\mu_{i}^{S}=\mu_{i}^{C,S}+\int p_{i}\left(\sigma \right)\cdot \mu_{i}\left(\sigma \right)d\sigma$$

This first term stands for a combinatorial contribution to the chemical potential and the latter signifies the residual partial molar Gibbs free energy. The COSMOtherm program utilizes generic formula [66,67,68] expressing combinatorial contributions to the chemical potential, which depend on the area and volume of all compounds in the mixture along with three adjustable parameters.

The practical calculations of these properties require a proper representation of the molecular structure. This is typically done by adequate exploration of the conformational space using COMSOconf for generation of the most energetically favorable structures. The actual computations were performed with the aid of TURBOMOLE rev. V7.5.1 interfaced with BIOVIA TmoleX 2021 (version 21.0.1), (BIOVIA, San Diego, CA, USA) using an RI-DFT BP86 (B88-VWN-P86) functional and def-TZVP basis set for geometry optimization and a def2-TZVPD basis set for single point calculations, the with inclusion of a fine grid marching tetrahedron cavity 90. Additionally, parameter sets with hydrogen bond interactions were included, and the van der Waals dispersion term was quantified using the “D3” method of Grimme et al. [69]. All of the calculations of the solubility were performed using COSMOtherm (version 20.0.0, revision 5273M), (BIOVIA, San Diego, CA, USA) [70] with BP_TZVPD_FINE_20.ctd parametrization.

Solubility computations of solid solutes were performed by iteratively solving the following equation:

(11) $$\log\left(x_{i}^{s,\left(1\right)}\right)=\frac{\mu_{i}^{p}-\mu_{i}^{s}\left(x_{i}^{s,\left(0\right)}\right)-\max\left(0,\Delta G_{fus}\right)}{RTln\left(10\right)}$$

where 𝜇_𝑖_^o^ is the chemical potential of a pure compound 𝑖, 𝜇_𝑖_^𝑆^,(i) represents the solute chemical potential generated after *i*-th iteration, and ∆𝐺_𝑓𝑢𝑠_ stands for solute Gibbs free energy of fusion. The zeroth order solution of Equation (11) corresponds to the solubility at infinite dilution 𝑥𝑖𝑆,∞ ≅ 1/𝛾∞ and is valid for small concentrations of the solute. For enhancing the accuracy, the obtained solubility is used for re-definition of the chemical potential and substitution into Equation (11) until self-consistency is achieved. It is evident that knowledge of the exact or approximated value of fusion Gibbs free energy must be provided outside of the COSMO–RS method, since it is dedicated to characterizing properties of bulk liquid phases.

#### 3.2.2. COSMO–RS–DARE Computations

The application of COSMOtherm for predicting properties of multi-component systems can be challenged by the fact that direct intermolecular interactions might eventually lead to new complexes formed, for example, between solute molecules or solute and solvent ones. These species are not typically included in the default definition of input files but might be important, since charge densities can be affected by the very fact of complex formation. This problems is addressed by COSMO-RS with dimerization, aggregation, and reaction extension (COSMO-RS-DARE) [71]. In this approach it is possible to extend the list of conformers of a given compound by inclusion of products of any reaction, in which the *σ*-surface is included for only one molecule and the other one is weighted out. This is done via “mcos” files, which have atomic weights set to one for atoms of the considered species and zero for atoms of the second interactions component. Hence, the number of atoms in such pseudo-conformers is the same as in monomers. It is essential to emphasize that interactions between *σ*-surface segments of these pseudo-conformers are not captured with the default settings of COSMO-RS and a new descriptor must be added to ensure that all new surface segments are included in computations. This is done by introducing two adjustable parameters in the formal form of the interaction Gibbs free energy between the *i*-th and *j*-th compounds:

(12) $$\Delta G_{ij}^{int}=H_{ij}^{int}-TS_{ij}^{int}$$

Hence, the COSMO–RS–DARE methodology relies on replacement of common interaction energy between surface segments, including the misfit, hydrogen bonding, and van der Waals forces by the above formula for interaction of two dimerization segments. The values of parameters of Equation (12) were estimated by fitting the computed solubility values to experimental ones for every temperature and solvent. The adaptation of COSMO-RS-DARE to solubility computations had been previously tested [72], and here the methodology was extended to also include binary solvents mixtures. This is done by treating of any the compositions of solvents as a distinct solvent. Moreover, in all COSMO-RS-DARE computations, only pairs formed by nicotinamide with itself or solvent molecule were considered, and only one interaction parameter was set for all such pseudo-conformers. The first simplification ignores contacts formed between solvent molecules and the justification for the second supposition comes from the assumption that electron density changes upon complex formation are of a similar magnitude.

Of course, in order obtain “mcos” files it is indispensable to perform conformational screening of bi-molecular systems. These bimolecular clusters were obtained by generating the most probable potential structures of the molecular complexes using the criterion of the highest contact probabilities between two molecules. The COSMOtherm program offers computation of contact statistics based on the probability of interactions between molecule surface segments, which were invoked by CONTACT = {1 2} ssc_probability ssc_weak ssc_ang = 15.0 command. Hence, the value of the dihedral angle between the two contacting molecules in the cluster was varied with a step size of 15° and the resulting geometries stored for further geometry optimization and clustering. The same scheme was adopted as for a typical conformation analysis done for monomers.

Highlighting the fact that the accurate computation of Gibbs free energies requires a more advanced level of theory, both the zero point energy (ZPE) and corrections for the electron correlation were taken into account. Hence, for proper accounting of the rotational and translational enthalpy in the gas phase of the thermal energy, the vibrational zero-point energy (ZPE) of all the species were computed on a BP/def2-TZVP level. The RI-MP2/def2-TZVPD ab initio approach was used for accounting of the electron correlation contributions. The obtained values were included in a standard thermodynamic cycle for computing values of Gibbs free energies of reactions between nicotinamide and solvent molecules. The computation of a chemical equilibrium was toggled by the keyword “reaction” in the COSMOtherm program.

## 4. Conclusions

Nicotinamide is an important representative of heteroaromatic amides functioning as a component of the coenzyme NAD. Since for majority of animals it is of exogeneous type, preventing nutritional deficiency requires supplementation through dietary intake. In general, knowledge of solubility is useful for designing and controlling the crystallization and extraction process, including the solvent selection, which is important in the case of obtaining and purifying natural compounds. In this paper five new aqueous mixtures of organic solvents were used to experimentally derive their solubility at temperatures ranging between 25 °C and 40 °C. It was found that the selected set of organic solvents offers all sorts of solvent effects, including co-solvent, synergistic, and anti-solvent features, enabling flexible tuning of the solubility. In the theoretical section, nicotinamide solubility was computed using two alternative approaches. The first one relying on default COMOS-RS computations failed in the confrontation with experimental values, due to highly inaccurate predictions of nicotinamide mole fractions in saturated solutions. It is argued that such a failure is to be addressed to the improper definition of the model of the studied solutions. Quantum chemistry computations at a sophisticated level of theory suggested the occurrence of stable complexes involving solute and solvent molecules. Augmenting of the model with only pairs formed by nicotinamide resulted in a spectacular improvement of solubility computations. However, although the application of COMSO-RS DARE allowed for perfect replication of the measured data, this was achieved at the cost of an additional single parameter for every system in the studied range of temperatures. The accuracy of such computations was found to be very high, which allowed for almost a perfect match between the computed and measured values. To the best our knowledge this paper reports for the first time the successful utilization of a COMSO-RS-DARE scheme for binary mixture solubility predictions.

## Figures and Tables

**Table 1:** Collection of melting temperatures and enthalpy of fusion values of nicotinamide determined in this work and reported in the literature. In the parentheses, the standard deviation values are provided (*n* = 3).

*T_m_* (K)	Δ*H_m_* (kJ/mol)
402.03 (±0.09) ^1^	24.21 (±0.27) ^1^
398.5 ^2^, 401.2 ^3^, 401.7 ^4^, 401.6 ^5^, 401.2 ^6^, 401.4 ^7^, 403.8 ^8^, 401.6 ^9^, 402.0 ^10^	16.7 ^2^, 23.7 ^3^, 22.58 ^4^, 20.5 ^5^, 25.4 ^6^, 23.2 ^7^, 23.8 ^8^, 25.5 ^9^, 26.94 ^10^, 23.9 ^11^

^1^ this work, ^2^ ref. [19], ^3^ ref. [44], ^4^ ref. [45], ^5^ ref. [17], ^6^ ref. [46], ^7^ ref. [47], ^8^ ref. [48], ^9^ ref. [49], ^10^ ref. [50], ^11^ ref. [43].

**Table 2:** Measured values of nicotinamide mole fraction (10^3^) in saturated solution of neat solvents. Ideal solubility was estimated using Equation (1) and DSC data provided herein.

T (K)	298.15	303.15	308.15	313.15
water	99.9 ± 5.1	114.9 ± 4.6	133.2 ± 2.4	154.2 ± 4.2
1,4-dioxane	16.9 ± 0.9	20.4 ± 0.8	24.6 ± 0.7	30.4 ± 0.6
DMSO	120.6 ± 3.5	150.7 ± 7.1	198.7 ± 3.2	265.5 ± 3.8
DMF	206.1 ± 11.9	226.7 ± 11.1	271.8 ± 9.3	325.9 ± 3.5
acetonitrile	8.6 ± 0.5	10.9 ± 0.5	13.4 ± 0.4	16.0 ± 0.2
methanol	74.1 ± 3.5	85.6 ± 2.5	100.1 ± 4.4	115.7 ± 2.6
ideal	118.12	133.08	149.60	167.82

**Table 3:** Measured values of nicotinamide mole fraction (10^3^) in a saturated solution of four binary aqueous solutions. x_2_ denotes the mole fraction of organic solvent in solute free solutions.

T (K)				
x_2_	298.15	303.15	308.15	313.15
acetonitrile				
0.2	112.9 ± 0.4	127.8 ± 3.8	144.0 ± 3.7	4.2 ± 165.7
0.4	101.1 ± 0.5	114.1 ± 4.6	132.3 ± 1.6	3.4 ± 152.0
0.6	64.8 ± 0.6	76.7 ± 1.9	92.0 ± 3.0	3.0 ± 109.0
0.8	40.6 ± 0.6	48.3 ± 1.7	57.6 ± 1.7	0.5 ± 69.5
1,4-dioxane				
0.2	91.6 ± 5.4	107.1 ± 4.2	125.6 ± 4.8	149.0 ± 1.7
0.4	84.6 ± 5.9	99.9 ± 4.3	118.5 ± 1.4	139.7 ± 3.0
0.6	71.4 ± 2.7	84.6 ± 3.5	103.6 ± 3.1	125.0 ± 1.4
0.8	49.3 ± 2.6	60.4 ± 0.3	75.3 ± 2.2	94.7 ± 2.9
DMF				
0.2	136.1 ± 3.7	157.6 ± 5.5	180.1 ± 2.5	214.5 ± 5.3
0.4	164.5 ± 7.8	187.1 ± 0.5	217.8 ± 8.1	256.4 ± 3.1
0.6	179.7 ± 3.0	202.5 ± 5.4	236.1 ± 2.5	287.5 ± 7.1
0.8	192.7 ± 4.8	216.1 ± 2.2	254.4 ± 5.8	312.4 ± 4.2
DMSO				
0.2	112.9 ± 2.5	129.2 ± 4.1	149.7 ± 1.5	185.4 ± 2.9
0.4	120.9 ± 5.9	139.2 ± 3.6	168.3 ± 4.8	217.7 ± 4.5
0.6	139.7 ± 5.9	170.8 ± 6.6	225.3 ± 4.4	304.3 ± 8.0
0.8	223.0 ± 4.5	254.6 ± 10.5	319.2 ± 4.4	402.7 ± 6.8
MeOH				
0.2	94.0 ± 0.8	112.0 ± 3.8	131.9 ± 3.7	152.0 ± 2.2
0.4	94.9 ± 0.6	114.3 ± 3.8	133.9 ± 3.5	156.8 ± 3.6
0.6	90.4 ± 5.0	109.1 ± 4.3	128.1 ± 4.6	151.0 ± 2.5
0.8	84.4 ± 3.0	101.4 ± 3.5	119.2 ± 1.3	141.0 ± 2.6

## Data Availability

The data used in this paper are available on request from the corresponding author.

## Supplementary Materials

The following are available online at https://www.mdpi.com/article/10.3390/ijms22147365/s1.
