# Supplementary material for: Thermodynamics and Intermolecular Interactions of Nicotinamide in Neat and Binary Solutions: Experimental Measurements and COSMO-RS Concentration Dependent Reactions Investigations
Source: Int J Mol Sci. 2021 Jul 8;22(14):7365. doi: 10.3390/ijms22147365 (PMC8306691; doi:10.3390/ijms22147365)
Supplement: Supplementary file 1 [file ijms-22-07365-s001.zip › ijms-1272305-supplementary.pdf]

Supplementary Material

Thermodynamics and Intermolecular Interactions of Nicotinamide in Neat and Binary Solutions: Experimental Measurements and COSMO-RS Concentration Dependent Reactions Investigations

Piotr Cysewski *, Maciej Przybyłek, Anna Kowalska and Natalia Tymorek

Department of Physical Chemistry, Pharmacy Faculty, Collegium Medicum of Bydgoszcz, Nicolaus Copernicus University in Toruń, Kurpińskiego 5, 85-950 Bydgoszcz, Poland; m.przybylek@cm.umk.pl (M.P.); 288310@stud.umk.pl (A.K.); n.tymorek@wp.pl (N.T.)

* Correspondence: Piotr.Cysewski@cm.umk.pl

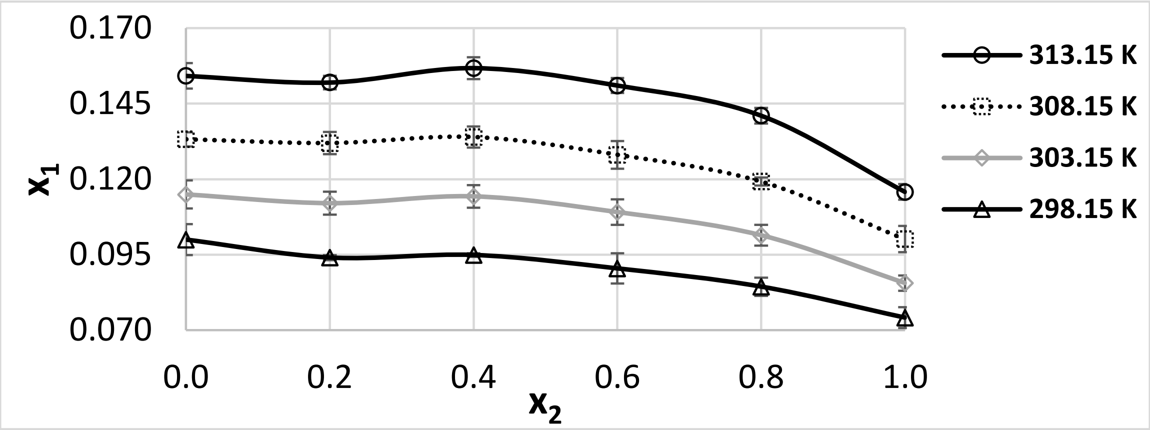

Figure S1. The solubility vs. temperature relationships of nicotinamide in methanol-water. The x_1, x_2 symbols denote molar fraction solubility and molar fraction of organic component in binary solvent, respectively. The error bares denote standard deviation values (*n* = 3).

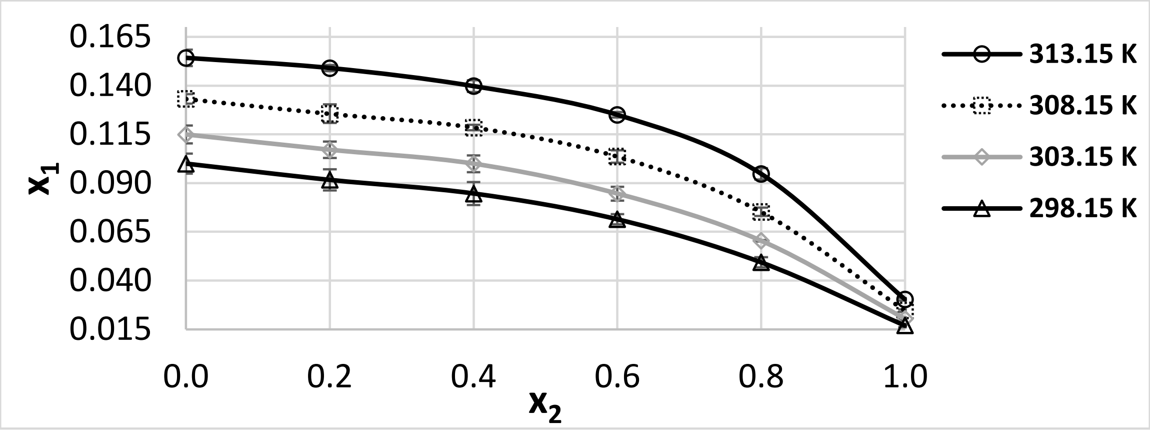

Figure S2. The solubility vs. temperature relationships of nicotinamide in 1,4-dioxane-water. The x_1, x_2 symbols denote molar fraction solubility and molar fraction of organic component in binary solvent, respectively. The error bares denote standard deviation values (*n* = 3).

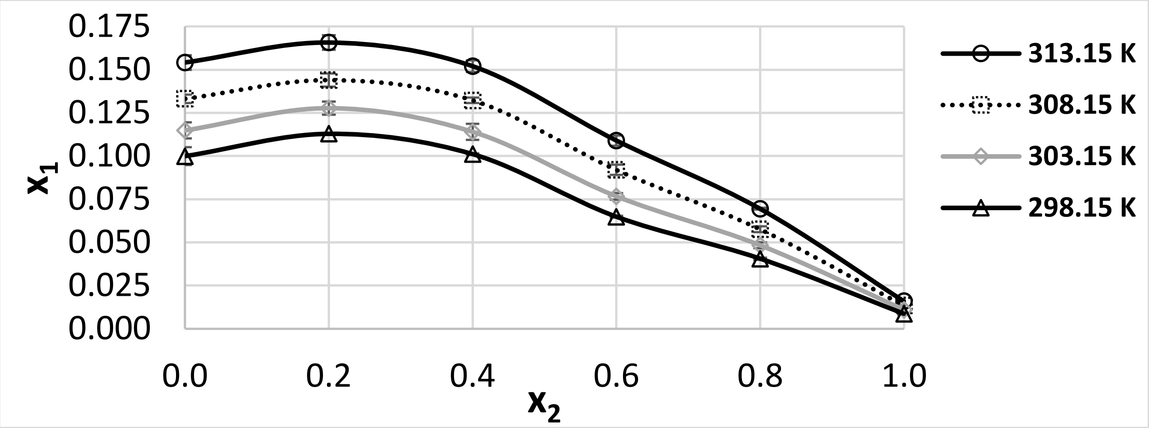

Figure S3. The solubility vs. temperature relationships of nicotinamide in acetonitrile-water. The x_1, x_2 symbols denote molar fraction solubility and molar fraction of organic component in binary solvent, respectively. The error bares denote standard deviation values (*n* = 3).

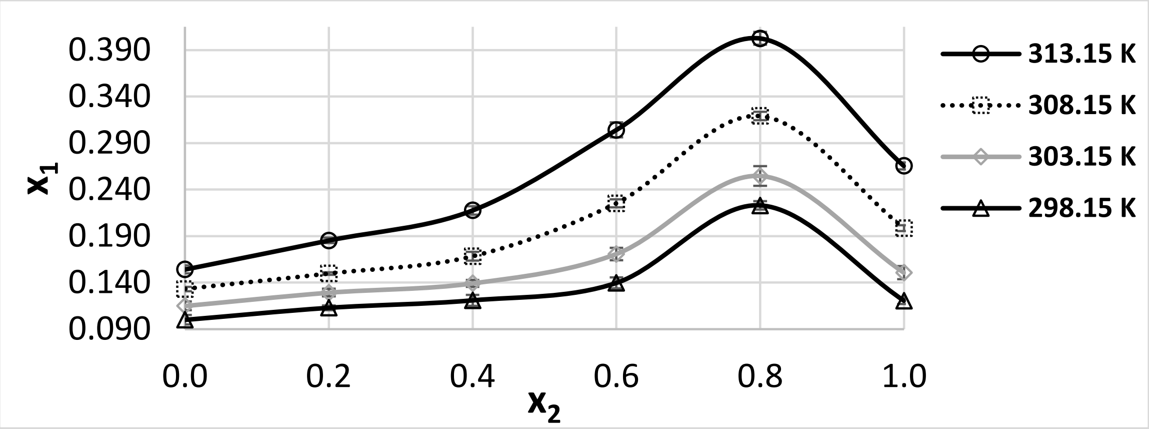

Figure S4. The solubility vs. temperature relationships of nicotinamide in DMSO-water. The x_1, x_2 symbols denote molar fraction solubility and molar fraction of organic component in binary solvent, respectively. The error bares denote standard deviation values (*n* = 3).

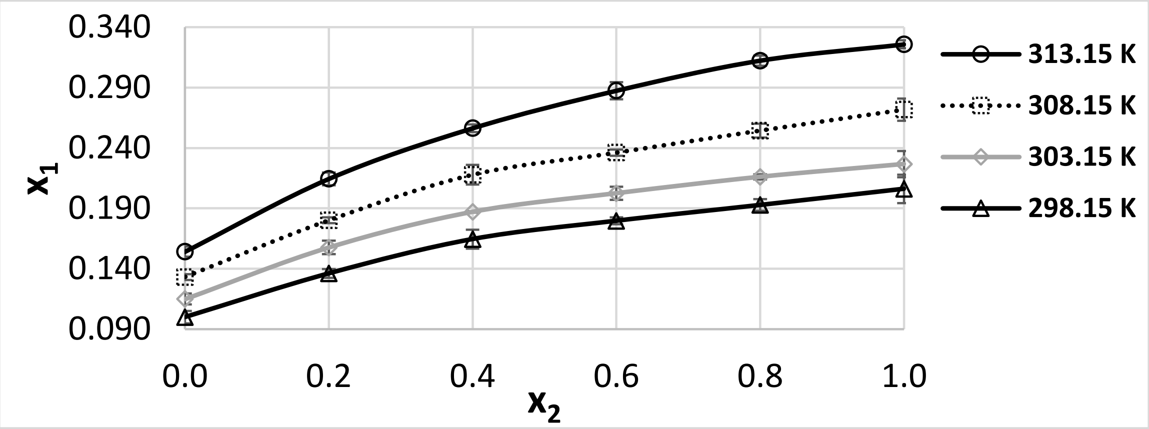

Figure S5. The solubility vs. temperature relationships of nicotinamide in DMF-water. The x_1, x_2 symbols denote molar fraction solubility and molar fraction of organic component in binary solvent, respectively. The error bares denote standard deviation values (*n* = 3).
